# Network structure of avian mixed-species flocks decays with elevation and latitude across the Andes

**DOI:** 10.1098/rstb.2022.0099

**Published:** 2023-06-05

**Authors:** Flavia A. Montaño-Centellas, Jenny Muñoz, Gabriela Giselle Mangini, Ian J. Ausprey, Felicity L. Newell, Harrison H. Jones, M. Elisa Fanjul, Boris A. Tinoco, Gabriel J. Colorado Z., Jennifer R. A. Cahill, E. Arbeláez-Cortés, Oscar H. Marin-Gómez, Pedro X. Astudillo, Esteban A. Guevara, Silvina Ippi, Molly E. McDermott, Amanda D. Rodewald, Erik Matthysen, Scott K. Robinson

**Affiliations:** ^1^ Instituto de Ecología, Universidad Mayor de San Andrés, La Paz, Bolivia; ^2^ Department of Biological Sciences, Louisiana State University, Baton Rouge, LA 70803, USA; ^3^ Biodiversity Research Center, University of British Columbia, Vancouver, Canada V6T 1Z4; ^4^ Instituto de Ecología Regional (IER) UNT-CONICET, Yerba Buena 4107, Tucamán, ‌Argentina; ^5^ Division of Conservation Biology, Institute of Ecology and Evolution, University of Bern, 3012 Bern, Switzerland; ^6^ Florida Museum of Natural History and Department of Biology, University of Florida, Gainesville, FL 32611, USA; ^7^ The Institute for Bird Populations, Petaluma, CA 94953, USA; ^8^ Instituto de Vertebrados, Zoología, Fundación Miguel Lillo. Facultad de Ciencias Naturales, Universidad Nacional de Tucumán, Yerba Buena 4000, Tucumán, Argentina; ^9^ Escuela de Biología, Universidad del Azuay, 010204 Cuenca, Ecuador; ^10^ Departamento de Ciencias Forestales, Universidad Nacional de Colombia, 050034 Medellín, Colombia; ^11^ Centro de Biodiversidad y Genética, Universidad Mayor de San Simón, Cochabamba, Bolivia; ^12^ Grupo de Estudios en Biodiversidad, Escuela de Biología, Universidad Industrial de Santander, 680002 Bucaramanga, Colombia; ^13^ Universidad Nacional Autónoma de México, Facultad de Estudios Superiores Iztacala, 45090 Tlalnepantla de Baz, México; ^14^ Colección de Ornitología, Programa de Biología, Quindío, Colombia, Universidad del Quindío, Armenia 630001, Colombia; ^15^ Laboratorio de Ecología, Universidad del Azuay, 010204 Cuenca, Ecuador; ^16^ Biodiversity and Conservation Biology, Swiss Federal Research Institute WSL, 8903 Birmensdorf, Switzerland; ^17^ INIBIOMA, CONICET-Universidad Nacional del Comahue, Quintral 1250, 8400 Bariloche, Argentina; ^18^ School of Environment and Natural Resources, The Ohio State University, Columbus, OH 43210, USA; ^19^ Cornell Lab of Ornithology and Department of Natural Resources and the Environment, Cornell University, Ithaca, NY 14853, USA; ^20^ Evolutionary Ecology Group, Department of Biology, University of Antwerp, 2610 Wilrijk, Antwerp, Belgium

**Keywords:** ecological networks, facilitation, mixed-species flocks, network modularity, species interactions

## Abstract

Birds in mixed-species flocks benefit from greater foraging efficiency and reduced predation, but also face costs related to competition and activity matching. Because this cost–benefit trade-off is context-dependent (e.g. abiotic conditions and habitat quality), the structure of flocks is expected to vary along elevational, latitudinal and disturbance gradients. Specifically, we predicted that the connectivity and cohesion of flocking networks would (i) decline towards tropical latitudes and lower elevations, where competition and activity matching costs are higher, and (ii) increase with lower forest cover and greater human disturbance. We analysed the structure of 84 flock networks across the Andes and assessed the effect of elevation, latitude, forest cover and human disturbance on network characteristics. We found that Andean flocks are overall open-membership systems (unstructured), though the extent of network structure varied across gradients. Elevation was the main predictor of structure, with more connected and less modular flocks upslope. As expected, flocks in areas with higher forest cover were less cohesive, with better defined flock subtypes. Flocks also varied across latitude and disturbance gradients as predicted, but effect sizes were small. Our findings indicate that the unstructured nature of Andean flocks might arise as a strategy to cope with harsh environmental conditions.

This article is part of the theme issue ‘Mixed-species groups and aggregations: shaping ecological and behavioural patterns and processes’.

## 1. Introduction

Mixed-species bird flocks (hereafter flocks), in which multiple species move and forage together, represent major facilitative interactions for forest birds. By joining these flocks, birds can benefit from increased foraging efficiency and protection against predators [1,2]. Individuals that join flocks also access social information from other flock members, particularly highly vigilant sentinel species, which enable them to exploit more exposed microhabitats and avoid predation [3–5]. Joining a flock, however, also results in costs for flocking species related to increased competition [6–8], as well as changes to foraging behaviour and foraging microhabitat to keep up with flock movement (i.e. activity matching; [4,9]). Given such potential trade-offs [1,10,11], individuals are expected to aggregate in flocks only if the benefits outweigh the costs. Because the benefits of flocking are context-specific and expected to vary with environmental conditions and flock mates [12–15], the composition, behaviour and structure of flocks (i.e. how species associate with each other) will change across environmental gradients, such as elevation [16–18], latitude [19] and human disturbance [12,20,21]. One proposed mechanism for these changes in flock structure is that the role of facilitative interactions in biological communities should increase with the harshness of environmental conditions [22–24] (i.e. the stress-gradient hypothesis [25]). Originally proposed for plant communities, this hypothesis has recently been tested among mobile organisms (e.g. [26,27]), but not yet in avian mixed-species flocks.

The Andes of South America is the longest mountain chain in the world and presents an ideal opportunity to assess how stressful environmental conditions influence the structure of flocks. In the Andes, environmental conditions change drastically over greater than 3000 m of elevation and 9000 km of latitude, affecting flocking behaviour and prevalence [16–19]. Species inhabiting harsher environments at higher elevations and latitudes, theoretically, have metabolic adaptions to low temperatures [28,29] that might cause subsequent increases in foraging rate and reductions in vigilance [30], two behaviours thought to promote flocking activity. Low temperatures can also reduce the activity and detectability of arthropod prey, further prompting birds to join flocks and increasing flocking propensity [31,32]. Flocking species inhabiting lower elevations and latitudes, on the other hand, are likely to face greater costs related to foraging competition [33] and activity matching (i.e. the changes to foraging and movement behaviour required to move with the flock [34,35]). The greater vertical segregation of microhabitats and expanded opportunities for partitioning of foraging niches [36–38], as well as greater year-round resource availability at lower elevations [36], might promote finer partitioning of foraging resources leading to the formation of multiple flock subtypes, often composed of species of similar body size [34,35] that share foraging stratum [35,39,40]. By contrast, higher elevation flocks in the Andes are often characterized by a mix of understory and canopy birds resulting from reduced canopy height and clear vertical strata in forest structure [41–43] and lack flock subtypes [18,44].

Changes in flock characteristics driven by the abiotic and biotic conditions across elevations have been formalized in the ‘open-membership hypothesis’ [14,44], which states that, in harsher and structurally simplified environments, interspecific flocking interactions are expected to be numerous and weak (i.e. reduced flock structure as species join and leave independently) and flocking aggregations are expected to be less exclusive (i.e. fewer preferred or avoided species associations, leading to a lack of clear flock subtypes). Consequently, flocks in harsher environments are expected to be more open and dynamic, whereas flocks at lower elevations are expected to form clear divisions into flock subtypes [14,44]). Although originally proposed along elevational gradients, predictions derived from the open-membership hypothesis might apply to structural changes in flocks across other gradients of environmental stress.

Because the temperate-to-tropical latitudinal gradient is characterized by increasing thermal stability and reduced environmental harshness [45,46], we would expect patterns similar to what we previously described for elevation [14,18]. The more seasonal climates towards temperate latitudes are known to affect participation in mixed-species flocks. For example, a greater proportion of the overall bird community participates in flocks in temperate systems relative to tropical ones [47], and at harsh temperate latitudes almost the whole forest passerine community participates in foraging flocks (e.g. [6,48]). In addition, the available feeding resources and foraging microhabitats are likely to decline in temperate forests in comparison to tropical ones [49]. The loss of specialized foraging microhabitats such as hanging dead vegetation, epiphytes and evergreen plants in temperate regions [49–51] might result in higher niche overlap [11,52] and lower activity matching costs in temperate flocking systems. Despite well-documented changes to flock richness, microhabitat diversity, seasonality of flocking behaviour and community participation with latitude [19,47,53], studies examining changes to flock structure across latitudes at a large scale are non-existent.

Besides elevation and latitude, local habitat characteristics also affect Andean mixed-species flocks. For instance, the loss of forest cover and increase in human disturbances can drive species loss, compositional turnover and changes in species associations [44,54,55]. The loss of continuous forest in Andean landscapes promotes the formation of smaller and less speciose flocks [12,54], likely because forest-specialist flocking species are lost from forest fragments [21,56]. Additionally, human disturbances may reduce vegetation complexity and the diversity of foraging microhabitats and feeding resources, directly affecting specialist insectivores [21,54] and the stability of flocking interactions [44]. Therefore, in habitats with reduced forest cover and reduced habitat quality, we expected flocks to be less structured, lacking a clear definition of flock subtypes, as described for disturbed and fragmented habitats in lowland systems [12,57].

Representing flocks as social networks can help us understand how environmental characteristics affect species associations and in consequence, shape group-level properties in flocks [58,59]. Networks of flocking species consist of nodes connected by edges, where nodes represent interacting species and edges represent the observed co-occurrences of species pairs in a flock [58]. Characteristics of such networks relate to (i) network connectivity (how flock interactions among species are organized and distributed within the network) and (ii) network cohesion (how aggregated species are within the network to constitute one unit). The open-membership hypothesis predicts fewer and more even interactions at higher elevations and latitudes, and thus, networks are expected to have increased connectivity and a consequent increase in network cohesion, with a preponderance of weak associations and low modularity. By contrast, flocking species at lower elevations and latitudes are expected to preferentially interact with a subgroup of species, creating ‘modules’ (flock subtypes) within the network, and therefore be more structured. In order to determine if the structural characteristics of flocks vary predictably across stress gradients, in this study, we use the open-membership hypothesis as a framework and examine changes to species association patterns of birds in mixed-species flocks.

In this study, we compiled a comprehensive dataset of Andean mixed-species flocks surveyed in six countries, across latitudes (10°N to 41°S) and elevations (400 to 4000 m.a.s.l.) to test predictions derived from the open-membership hypothesis (table 1). Specifically, we examine how the overall structure of interaction networks of flocking species, as quantified by network-level metrics related to flock connectivity and cohesion, varies across four major environmental gradients in the Andes: latitude, elevation, forest cover and human disturbance. We expected (i) flock richness and network structure to decrease with increasing levels of stress (high elevation and latitude, low forest cover and high levels of human disturbance), (ii) network connectivity and cohesion to increase at higher elevations and latitudes, and to decrease in structurally complex habitats with high forest cover and low levels of disturbance. Finally, because both the increased benefits (stress facilitation) and reduced costs (activity matching and competition) of flocking at higher levels of stress should result in higher participation across species with mostly weak and non-exclusive associations [14], we expect (iii) Andean flock composition at higher latitudes and elevations to be mostly driven by non-social factors (i.e. to reflect random interspecific associations), resulting in open-membership associations. Our hypotheses and the mechanisms driving expected patterns are summarized in table 1.

**Table 1.** Predictions of the open-membership hypothesis about changes in the structure of Andean mixed-species flocks across elevation, latitude and gradients of forest cover and human disturbances.

environmental gradients	mechanisms	ecological outcome	predicted pattern
elevation and latitude	Abiotic and biotic filtering act upon avian assemblages shaping community structure.	Flocking assemblages are structured by community assembly forces and therefore changes in flock richness are reflective of those in the whole community.	Species richness decreases with increasing elevation and latitude.
	Harsher abiotic conditions and less climatic stability impose greater stress on individuals; thus, greater foraging benefits can be obtained by facilitation at higher elevations and latitudes.	Flocking behaviour is more prevalent in extreme abiotic conditions because (i) higher benefits can be gained by facilitation where energy demands are higher; (ii) facilitation benefits related to foraging efficiency might be higher in areas where resources are depleted and/or unavailable.	Flocks at extreme elevations and latitudes are more dynamic and unstructured, with a more open membership and less specialized interactions, characterized by high values of network connectance, average degree and clustering, and low values of modularity.
	Milder abiotic conditions and more climatic stability impose less stress on individuals, and result in higher levels of specialization, fine partitioning of foraging niches and increased levels of competition. Thus, fewer foraging benefits can be gained, and higher costs of joining a flock might occur at lower elevations and latitudes.	Interspecific interactions are more specialized in mild abiotic conditions because (i) gained benefits to not outweigh the costs of activity matching and the potential costs of competition, and (ii) facilitation benefits related to foraging efficiency are likely not higher in areas where resources are abundant/available.	Flocks at lower elevations and latitudes are less dynamic and structured (have more subtypes) with a more restricted membership, characterized by low values of network connectance, average degree and clustering, and high values of modularity.
forest cover and human disturbances	Reduced forest cover and increased human disturbances (i.e. low quality habitat) result in the local extirpation of flocking species, in particular specialist insectivores.	Flocks in disturbed and more fragmented areas will be composed mostly of generalists, lacking foraging specialists.	Greater species richness per flock with increasing forest cover and decreasing human disturbances.
	Greater number of available foraging niches and feeding resources in undisturbed and more continuous forests (i.e. high quality habitat) allows for finer partitioning of foraging strata and microhabitats among members of mixed-species flocks, increasing costs related with activity matching.	Greater habitat quality (i.e. areas with high forest cover and low levels of disturbance) harbours more structured flocks, with a cleared differentiation of flock subtypes (i.e. canopy and understory).	Flocks in undisturbed areas and areas with high forest cover will be overall less connected and cohesive, characterized by low values of network connectance, average degree and clustering, and high values of modularity.
	Reduced number of available foraging niches and food resources in disturbed and fragmented forests (i.e. low habitat quality) will benefit opportunistic and generalist species, reducing costs related to activity matching.	Poorer quality habitat (i.e. areas with low forest cover and high levels of human disturbance) harbours less structured flocks that lack a clear differentiation of flock subtypes.	Flocks in disturbed areas and areas with low forest cover will be overall more connected and cohesive, characterized by high values of network connectance, average degree and clustering, and low values of modularity.

## 2. Methods

### (a) Mixed-species flocks data collection and selection criteria

We gathered data on Andean flocks from published and unpublished sources. First, we conducted a broad literature search on Google Scholar with the terms ‘mixed-species flock’ or ‘*bandadas mixtas*’ and ‘Andes’. No temporal restrictions were applied to our search. We pre-selected all relevant publications and inspected them to see if they had available data. In many cases, composition data of individual flocks (necessary to construct an interaction network) used in these publications were unavailable and, thus, we contacted the author(s) to request this information. A dataset was included in our analyses only if (i) the study was conducted within terrestrial habitats on the Eastern Andean slope and/or the immediately contiguous lowlands and (ii) a flock was defined as an aggregation of individuals of at least two species that move together while foraging in the original study [6,60,61]. Similarly, we (iii) only included data collected using the ‘gambit of the group’ method, where all species observed within a single flock are assumed to be associating and are assigned reciprocal ties in the network [58,62]. Thus, in our analyses, we did not include aggregations of frugivorous species which form in response to clumped resources and are not mobile associations. We further selected studies with greater than or equal to 10 surveyed flocks per site to construct social networks, a minimum required to adequately describe a complete network when using gambit of the group sampling [63,64] to measure fluid social interactions such as those among flocking species [64]. If a study compared flocks at more than one site and/or across seasons (e.g. [32,44]), each site and season combination was included as an independent dataset following each author's criteria (i.e. if the original author considered these as two independent datasets in the original manuscript, because of a complete turnover of species, for example). We updated and standardized the taxonomy for all the studies following the most recent taxonomy from BirdLife International [65]. Our final dataset included the species composition of 3676 independent flocks surveyed between 1976 [39] and 2019 [55], and organized into 84 independent datasets (hereafter referred to as sites) across the Andes and adjacent lowlands (greater than 400 m). A list of data sources is available in the electronic supplementary material.

### (b) Interaction networks of flocking birds

**Figure 1.**
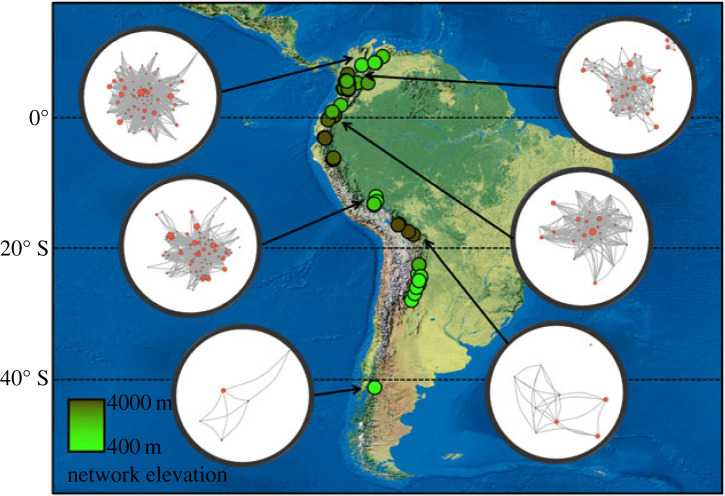
Distribution of mixed-species flock networks observed across the Andes (*N* = 84). Dots on the map are colour coded according to the elevation of the network (in m.a.s.l.). Examples of networks from relatively undisturbed forests are displayed on the left for lower elevations (north to south: C1, LowlandsTF, Patagonia) and on the right for higher elevations (north to south: North Antioquia, Guandera, Sacha Loma).

We generated a weighted network for each site (*N* = 84; figure 1) using presence–absence, flock-by-species matrices [58,66]. Weighted networks are more informative in ecological studies assessing the preference or avoidance of species pairs [67]. The strength of species co-occurrences in the networks was quantified using the simple ratio index (SRI), an undirected weighted measure that describes the probability that two species are observed together [66]. For each pair of species, *a* and *b* in a network of mixed-species flocks,

$$\mathrm{SRI}=\frac{x}{\left(y_{a}+y_{b}+x\right)},$$

where *x* is the number of flocks where *a* and *b* were observed together, *y_a_* is the number of flocks where *a* was observed but not *b*, and *y_b_* is the number of flocks where *b* was observed but not *a*.

To examine if species associations within observed networks are caused by preferential assortment among species (preference or avoidance of specific flock mates) and not by non-social aspects (i.e. local species densities), we constructed null models by permutating raw observational data for each network [68]. In each permutation, the observations of species among flocks are swapped within the matrix and thus, species richness and species incidence are retained [68]. We conducted 3500 permutations per site; in each step of the permutation process, two species are randomly selected from two flocks in which they do not co-occur and swapped, following the algorithm proposed in Farine & Whitehead [58]; then, a null network is created with the new co-occurrence matrix.

To measure the degree of structuring in each network, we calculated the network coefficient of variation (CV: the standard deviation of the edge weights divided by the mean of the edge weights); the most easily interpretable metric of the variability of the associations within a network [59]. We compared the observed CV of each observed network with the distribution of CV values of the 3500 random networks. A value of CV larger than 95% of the values of random CV suggests that the observed network contains more preferred/avoided relationships than expected. *p*-values were calculated by taking the number of times the CV values from the random networks were larger than the values from the observed network, divided by the number of permutations [68].

To characterize network connectivity and cohesion, we used four network-level metrics (electronic supplementary material, table S1). Network connectivity is a representation of how the nodes are connected within the network, and it was assessed with (i) normalized average degree, a measure that represents the average of the number of edges for each node, divided by the total number of nodes minus one; and (ii) network connectance, the number of observed links, divided by the number of possible links between all pairs of species. These two measures can be interpreted biologically as the number of overall flocking co-occurrences and the average of species-specific links, respectively (electronic supplementary material, table S1). Network cohesion describes how unified networks are, and was quantified using two metrics: (iii) network modularity, a measure of the strength of division of a network into modules or highly connected blocks of species in the whole network, was quantified with a modularity optimization method (index Q; [69]), using Clauset *et al*.'s [70] algorithm for community detection; and (iv) network global clustering index [71], a metric that refers to the degree to which all nodes in a network tend to cluster together. Biologically, modularity can be interpreted as the occurrence of flock subtypes within a system, typically differentiated by foraging stratum or body size. Clustering, on the other hand, gives information on the grouping of nodes with their neighbours and therefore tends to be negatively correlated with modularity [72]. All analyses were performed in R [73]. We used functions in packages *igraph* [74], *tnet* [75] and *asnipe* [76] to calculate network-level metrics and to visualize networks.

To test whether network-level metrics were significantly different from the null model expectation, we calculated them for each randomized network as described above. We then compared observed values with the distribution of values obtained from the randomized networks. We considered the network-level metric significantly different from random if it was lower than 0.025% or higher than 0.975% of the values obtained from random networks [68].

### (c) Environmental predictors of network structure

We gathered geographical coordinates and elevation for each site (network) from the original publication or obtained them directly from the authors. If coordinates and elevation information were provided for each flock, we used the averaged values to characterize the geographical location and elevation for each site. Similarly, we used flock-specific coordinates to extract data on remotely sensed forest cover and human disturbance and averaged these values per site. Anthropogenic disturbance for each site was described with the human footprint score proposed by Venter *et al.* [77], which combines information on the extent of built environments, croplands and pasturelands; human population density; night-time light pollution; and density of railways, roads and navigable waterways, between 1993 and 2009, a range of years where roughly two-thirds of the sites included in our analyses were surveyed. Forest cover was extracted from the Global Forest Watch dataset v1.9 which calculates vegetation greater than 5 m in height at a 30-m pixel resolution based on Landsat data [78]. For analysis, we used 2019 forest cover, which was the last year in which flock data were collected. Using the original coordinates for each independent flock, we calculated the per cent of pixels with greater than or equal to 50% forest cover within a 500 m radius buffer, and then averaged these values across flocks within a network. Because of natural fragmentation in high-elevation forests dominated by *Polylepis* spp. (greater than 3500 m.a.s.l.), the Global Forest Watch dataset provided a poor measure of vegetation cover, and thus, vegetation polygons were manually digitized and averaged across coordinates for the three networks in *Polylepis* forests using high-resolution imagery from Google Earth to calculate per cent cover within a 500 m radius.

### (d) Network structure across environmental gradients

To examine the effects of elevation, latitude, forest cover and human disturbance on the observed properties of the networks, we used generalized linear models (GLMs) and Beta regression models. Because network-level metrics across networks were quantified with standardized metrics (electronic supplementary material, table S1), they are comparable across sites. For species richness and covariance, we used GLMs assuming Gamma distribution errors because both response variables are continuous and positive. For modularity and connectance, we used GLMs assuming Gaussian error distributions. We opted to log-transform connectance before analyses because the observed values were bounded between 0 and 1, but their distribution was heavily right-skewed. Finally, for average degree and clustering, we used Beta regression models because both variables are bounded between 0 and 1 [79].

Prior to analysis, we examined correlation coefficients among variables; all correlation coefficients were less than 0.5 (electronic supplementary material, table S2), so we included all four variables as predictors. In addition, we included the interaction between latitude and elevation in our regressions and retained it in our final models if significant. Finally, we calculated variance inflation factors (VIFs) for each predictor in our models to detect collinearity. All VIF values were relatively low (1.65 for elevation, 1.49 for latitude, 1.45 for forest cover and 1.31 for human footprint) further supporting our decision of including all four predictors.

As in any inferential study, we used a set of repeated samples (a set of flocks) to represent the true interspecific association patterns in flocks. Thus, differences in sampling effort and network size can potentially become confounding factors when comparing networks. To account for this, we examined the correlations among the number of nodes per network (number of flocking species) and the number of flocks used to create each network (as a measure of effort) with our environmental variables. In all cases, correlations were weak (electronic supplementary material, table S2), indicating that flocks were adequately sampled. To further test the robustness of our results, we repeated all analyses with two more conservative subsets of data. The first one included (i) only networks with 20 or more flocks per site (*N* = 72 sites) and the second subset included (ii) all networks except the three in *Polylepis* forest sites (for which forest cover was calculated manually, as explained above). With few exceptions, the overall trends of results and major conclusions did not change when using these subsets of data. We therefore present results for all 84 sites and provide calculations for data subsets in the electronic supplementary material, figures S1 and S2.

Finally, the relationship between sample effort and the robustness of network-level metrics is complex and dependent on the nature of the ‘true’ network structure itself [80]. Although we cannot know *a priori* the full structure of the true network association patterns at each of our sites, we used the method proposed by Shizuka & Farine [80] to quantify a measure of the robustness of our modularity metrics. This method calculates the assortativity index (r_com), a measure of the level of confidence in derived network metrics based on the detectability of associations. Values for r_com ranged between 0.31 and 0.96, similar to those reported for mixed-species flocks in Shizuka & Farine [80]. Using the r_com values, we further subset our data and performed regressions on network modularity including only those networks with r_com > 0.4 (*N* = 50). Results are presented in the main text and in the electronic supplementary material, tables S5 and S6. Unfortunately, no similar indices have yet been suggested to quantify for the robustness of other network metrics used in our study, and thus we limited this analysis to modularity.

## 3. Results

### (a) Interaction networks of flocking species

**Figure 2.**
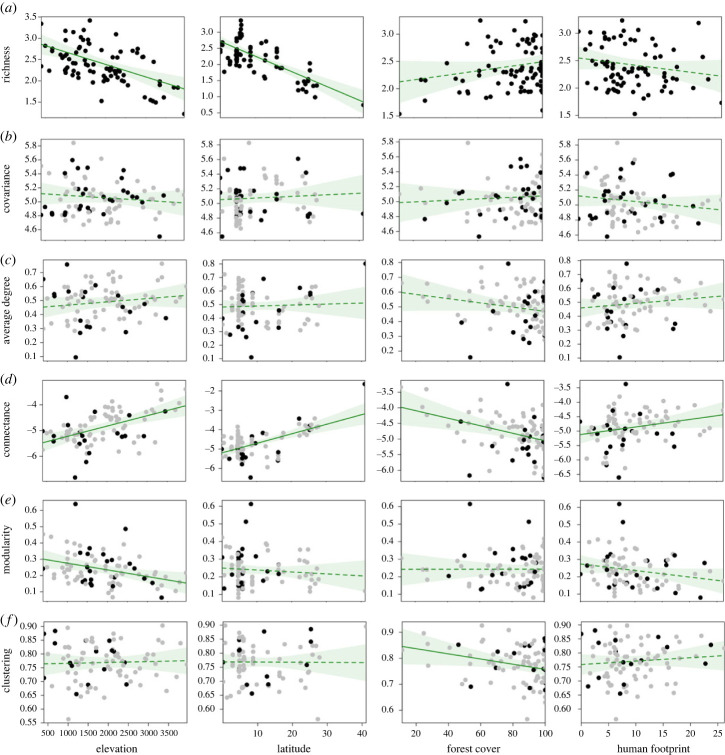
Relationship between environmental predictors (elevation [in m.a.s.l.], absolute latitude [in decimal degrees], forest cover and human footprint) and structural characteristics of interaction networks of flocking species. (*a*) Average species richness per flock, (*b*) network covariance, (*c*) average degree, (*d*) connectance, (*e*) weighted modularity and (*f*) weighted clustering. Lines represent partial regressions from the models described in the electronic supplementary material, table S3; complete lines are used for significant predictors and dashed lines for non-significant predictors. Dots represent expected values for each network property as a function of each predictor, while all other predictor variables are held constant. Dark dots are network structural metrics that differ from random expectations and grey dots are metrics that do not differ from expectations if flocking occurs at random.

Species richness per network ranged between 5 and 112 (Patagonia in Argentina and Jiri in Bolivia, respectively) with a range of 2.5 to 24.2 mean richness per flock (Patagonia in Argentina and Cocha Cashu in Peru; electronic supplementary material, table S3). Species richness per network and the average number of species per flock were poorly correlated with the number of flocks in each network (Pearson's moment correlation = 0.34 and Pearson's *r* = 0.04, respectively; electronic supplementary material, table S2). We found significant social structure (network CV significantly higher than the null expectation) in only 29 sites (34% of all networks), indicating that Andean flocks are mostly unstructured and dynamic (open). Sites with significant values of network CV were distributed across latitudes and elevations (figure 2*b*).

The four network-level metrics describing connectivity and cohesion varied greatly among networks. Average degree ranged from 0.14 to 0.8, and connectance (log scaled) ranged from 0.002 to 0.16 (electronic supplementary material, table S3). Overall, the most connected networks were mainly located at extreme latitudes and elevations including Patagonia in Argentina, Cajas NP in Ecuador and Sachaloma in Bolivia (the latter two greater than 3500 m.a.s.l.). Weighted modularity ranged from 0.07 to 0.64 and weighted clustering from 0.56 to 0.93 (electronic supplementary material, table S3). Most of the highly clustered networks were at tropical middle and high elevations (e.g. Guandera RF in Ecuador, Fredonia and El Silencio in Central Colombia, and Sachaloma in Bolivia, all above 2000 m.a.s.l.), but the network in Patagonia was also strongly clustered (value = 0.90). The most modular networks were located at lower elevations in Peru (Lowlands B and Lowlands TF, approx. 400 and 600 m.a.s.l.) and mid-elevations in Colombia (e.g. North Antioquia, approx. 2400 m.a.s.l.). Overall, network-level metrics differed from those expected from random association patterns only in 25% or fewer of the networks (figure 2*c–f*), further supporting the idea that Andean flocks are mostly unstructured across a variety of stress gradients.

### (b) Predictors of network structure and network properties

The average species richness per flock increased towards lower latitudes and lower elevations (figure 2; electronic supplementary material, table S4). The interaction between latitude and elevation was positive, indicating that species richness per flock decreases more rapidly with elevation at tropical latitudes. None of the predictors explained differences in overall network structure measured as network covariance (figure 2; electronic supplementary material, table S4).

As predicted by the open-membership hypothesis, there was a trend of increasing network connectivity (average degree and connectance) with elevation and latitude, but these relationships were only significant for connectance (figure 2; electronic supplementary material, table S4). The interaction between elevation and latitude was negative in both models, implying that the decay in connectivity at lower elevations is more pronounced in tropical latitudes. Overall, we found network connectivity to decrease at higher levels of forest cover and increase with increasing levels of human disturbance (figure 2; electronic supplementary material, table S4). Also consistent with our predictions, there was a trend of increasing network cohesion (weighted clustering and modularity) with higher levels of environmental stress. Network clustering significantly decreased with increasing forest cover, with highly clustered networks in more open habitats. Network modularity, on the other hand, decreased with increasing elevation, with clearer detection of flock subtypes (higher modularity) at lower elevations (figure 2; electronic supplementary material, table S4). Although latitude had a negative effect on both modularity and clustering, neither of these relationships were significant (electronic supplementary material, table S4).

**Figure 3.**
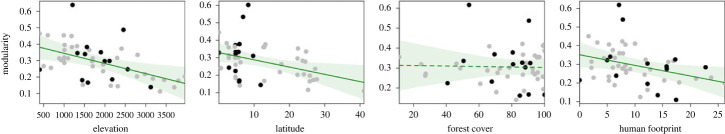
Relationship between environmental predictors (elevation [in m.a.s.l.], absolute latitude [in decimal degrees], forest cover and human footprint) and weighted modularity, when considering only modularity values with community assortativity values (i.e. robustness index) above 0.4 (*N* = 50). Lines represent partial regressions from the model described in the electronic supplementary material, table S6; complete lines are used for significant predictors and dashed lines for non-significant predictors. Dots represent expected values for each network property as a function of each predictor, while all other predictor variables are held constant. Dark dots are network structural metrics that differ from random expectations and grey dots are metrics that do not differ from expectations if flocking occurs at random.

Overall, similar patterns were detected when analysing data subsets that included only networks with at least 20 flocks (electronic supplementary material, figure S1) and excluding sites within *Polylepis* forests (electronic supplementary material, figure S2). However, when using a third subset of data that included only networks with robust estimations of modularity (networks with community assortativity indexes r_com > 0.4), we not only detected the similar negative effect of elevation on modularity, but the negative relationship of latitude was also significant (figure 3; electronic supplementary material, table S6), suggesting network modularity reduces both at high elevations and latitudes.

## 4. Discussion

We documented changes in the richness and structure of networks of mixed-species flocks across four main environmental gradients in the Andes: elevation, latitude, forest cover and human disturbance. We found support for our predictions based on the open-membership hypothesis: mixed-species flocks with fewer and more even interactions at higher elevations and latitudes (table 1). Furthermore, our results suggest that, although elevation was the strongest correlate of structural properties of avian flocks in the Andes, forest loss also influenced network-level attributes, with less connected and less cohesive networks in sites with higher forest cover.

### (a) Interaction networks of mixed-species flocks in the Andes are largely unstructured

In support of the open-membership hypotheses, we found that roughly two-thirds of the networks did not have greater structure (measured as network CV) than the null expectation, suggesting that species within these networks might associate opportunistically, leading to numerous pairwise associations and a context-dependent species composition of flocks. In general, we also found that network metrics related to both connectivity and cohesion were not different from those expected by random associations within the flock, and networks without structure were widespread across elevations and latitudes (figure 2). The reduced structuring in Andean flocks has been frequently documented, with great variability in flock size and richness even at local scales [18,41,42,44]. Species dynamically join and leave Andean flocks as they enter and exit their territories [41,81], increasing the variability of flock composition. Notably, our findings contrast Moynihan's [82,83] early descriptions of Andean flocks, where flocks are described as highly structured even to the point of favouring the convergent evolution of plumages as a form of social facilitation. We argue that the steep topography, and frequent disturbance by landslides, in Andean landscapes reduce the vertical segregation of vegetation and thereby play a key role in preventing the formation of stratum-specific flock subtypes and additional coevolved structuring of flocking interactions. The canopies of Andean forests are typically shorter (approx. 20 m) than those of lowland forests (approx. 30 m), and on steep slopes the canopy and understory are only a few metres apart and vertical stratification is greatly reduced in comparison to the well-segregated forest strata in the contiguous lowlands [36]. Furthermore, frequent landslide disturbances on steep slopes help maintain patches of early- and mid-successional vegetation on the landscape and increase the horizontal heterogeneity of habitat structure [36,84]. By contrast, lowland forest strata are more discrete, resulting in segregated foraging microhabitats for canopy and understory flocks that greatly increase the costs of activity matching [9] and could decrease the benefits of social information [85].

A strong correlation between the species richness and composition of the flock-joining and full Andean bird communities has been described [14,17], with elevational changes in flocks mirroring elevational changes in whole avian assemblages. Consistent with these observations, we found that flock species richness decreased with both elevation and latitude in a similar fashion as whole avian communities [86]. Altogether, our findings imply that Andean mixed-species flocks are overall dynamic and unstructured across latitudes and elevations. Furthermore, similar findings of elevational changes in flock characteristics (species richness, compositional turnover and flock organization) have been made in other montane systems (e.g. [87–90]), suggesting montane flocks in other regions of the world might also be more dynamic and open than their lowland counterparts. Further studies should focus on testing the generality of the open-membership hypothesis in other mountain ranges.

### (b) Network connectivity and cohesion increase with latitude and elevation

Our analyses showed consistency in the direction of latitude and elevation effects on network structure. Overall, network connectivity and cohesion increased with both elevation and latitude, with significantly higher connectance (figure 2*d*) and lower modularity (e.g. reduced partitioning of associations into flock subtypes; figures 2*f* and 3) in temperate regions and at higher elevations. Altogether, our results extend the generality of the results of previous studies of elevational [18] and latitudinal effects [19] on Andean flocks' structure.

Our findings are consistent with the stress-gradient hypothesis, where the role of facilitative interactions in biological communities increase at higher levels of environmental stress [22–24]. In more stressful and less vertically stratified environments (i.e. higher elevations and latitudes), the costs of activity matching become less important, and flocks exhibit numerous loose connections with no discernible subtypes. This contrasts with lower elevations and latitudes, where species show higher levels of foraging specialization that increase the costs of activity matching [33]. To reduce these costs and obtain the benefits of relevant social information about predators and feeding resources, species need to aggregate with flock mates that share a similar foraging stratum, movement speed and phenotypic characteristics [51,91], resulting in more discrete flock subtypes. Accordingly, we found a strong negative effect of elevation on network modularity (figure 2). Importantly, the negative effects of elevation and latitude on network-level properties held when using a more conservative subset of data (networks with 20 or more flocks) and became even stronger when examining a subset of data that included more robust estimations of modularity (figure 3; electronic supplementary material, table S6). Unfortunately, we are not aware of similar robustness index for the other network-level metrics included in our analysis and, thus, we can only draw conclusions on the strong negative effects of elevation and latitude on weighted modularity. Nevertheless, the similarity between the overall trends we detected when using different subsets of data suggests that, although there might be noise created by site-level differences in sampling protocols that compromised our ability to detect more significant relationships, future research that controls for sampling inconsistencies will likely find similar trends to those we describe. Collectively, our findings add to the growing body of evidence supporting the stress-gradient hypothesis (i.e. that the propensity of facilitative interactions should increase with increasing levels of environmental stress; [22–24]) as a mechanism driving network structure in mobile animals that have much greater freedom of choice in species interactions that sessile plants [26,92], while suggesting that activity matching plays an important role structuring flocking networks at lower elevations and latitudes.

### (c) Habitat quality influences flock structure

Besides climatic stability, we hypothesized that habitat characteristics at a local scale, represented by forest cover and human disturbance, would also affect flock properties. Human disturbance generally decreases the richness and size of flocks [47], and studies on fragmented landscapes also show a decay in network properties (i.e. a loss in structure and complexity) with increasing levels of fragmentation [12,57], and human disturbance [13,20,44,54]. Consistent with these observations, we found more structured networks (low connectivity and cohesion) at higher levels of forest cover and less structured networks in more disturbed habitats (figures 2 and 3).

The differences in network-level metrics of flocks across gradients of forest cover and human disturbances might relate to differences in the costs of activity matching along these gradients. The costs of activity matching are expected to be higher in more structurally complex habitats [14], such as those with continuous and undisturbed forests. In these relatively ‘good quality’ habitats, foraging microhabitats may be more diverse [93] and, therefore, more finely partitioned among flocking species which are often specialized on specific foraging substrates [49,94] and foraging height bands [50,95]. In consequence, species are more likely to encounter their specific foraging microhabitat and avoid most costs of exploitation competition from flock mates [44]. Indeed, the functional diversity of foraging behaviours and substrates of flocking species was found to increase with vertical vegetation structure in Andean forests [21]. The loss of habitat quality, on the other hand, might affect the local abundance, and in consequence, the relative importance of forest-dependent species within flocks [18,44]. For example, species in Andean flocks preferentially associate with ecologically similar ‘nuclear’, or leader species [91], and in disturbed and fragmented sites the core ‘nuclear’ role is more often played by omnivorous and edge-associated tanagers (e.g. genus *Tangara*; [44]). These changes to flock leadership, and the broader changes to habitat, may allow more edge- and open-habitat species to occasionally participate in flocks alongside forest birds, increasing connectivity. In summary, our results suggest that changes in forest characteristics are likely to affect the cost–benefit balance species incur when joining a flock, resulting in differences in the structure of the interaction networks with more structured flocks in continuous and undisturbed habitats.

Additionally, flock structural characteristics might relate to the differences in predation risk in different habitat types [96]. Avian flock structure, modularity and organization might also be determined by the abundance and diversity of predators (e.g. raptors in the genera *Accipiter* and *Micrastur*; [97]). In more predator-rich environments, the need to gain social information related to predator type and location (i.e. to understand the message conveyed by flock mates, particularly sentinel species) increases. Moreover, if predation benefits are obtained by mechanisms such as the dilution effect, or the confusion effect [98], having phenotypic similarities with flock mates (i.e. not being the flashier, larger or slower individual in the group) might also be favoured. In consequence, flocks in predator-rich environments would be expected to have strong modularity with highly coevolved interactions among similar species that share the same predators and movement rates. Conversely, the dynamic compositional changes of flocks both at higher altitudes and in disturbed environments could reflect a relaxation of predation pressure. For instance, the diversity and local abundance of specialized bird-eating raptors can be negatively affected by human disturbances in the Neotropics [99,100]. If greater predator richness or density correlates with greater predation risk for flock-joining species, flocks in areas with greater forest cover and lower levels of human disturbances should be more structured and composed of more similar species. Because species that share predators may have similar body sizes and foraging strategies, it is possible that both mechanisms (i.e. avoiding activity matching costs and reducing predation risk) operate in driving changes to flock organization across gradients of habitat quality.

### (d) Environmental gradients and the open-membership hypotheses

We present strong evidence in support of the open-membership hypothesis, suggesting that flocks across the Andes are primarily dynamic [41]. The degree of flock openness is, however, not homogeneous across environmental gradients, and likely represents a continuum with higher values in harsher conditions, where flocks may be composed of more generalist and omnivorous species [14]. Such species, particularly gregarious tanagers, may more easily facultatively join and leave flocks, often leaving to forage in single-species groups on fruit or nectar [101], and suffer fewer activity matching costs due to the simplified structure of high-Andean forests. Our observations suggest that montane flocking species across the Andes might use dynamic flocking as a strategy to cope with unstable conditions and scarce resources, having the option to either join the flock or not depending on the environment through which the flock moves. In more stable conditions, towards lower elevations and tropical latitudes, flock subtypes emerge and networks become more structured, with a clearer separation among canopy and understory flocks.

We argue that across elevations and latitudes, environmental conditions along mountain ranges are not conditions that would favour elaborate coevolutionary interactions among flocking species such as those postulated by Moynihan [82,83]. Although our findings imply that harsher environments clearly favour flocking, defences such as group vigilance, dilution of predation risk and the selfish herd [98], which work regardless of the roles played by the component species, are likely to be the main benefits for participant species. Crucially, however, the costs of competition and activity matching may be significantly reduced at high elevations due to reduced exploitation competition (lower flocking species richness, diet supplementation with fruit) and simplified vegetation structure. The complex systems of eavesdropping and sentinels that have been well documented in the tropical lowlands of South America (e.g. [5,102]) do not seem to apply to Andean flocks. Perhaps the relatively young age of the Andean range, reduced vertical stratification resulting from its steep topography and frequent landslide disturbance, and continual succession dynamics all play against the evolution of complex specialized flocking structure attained in the adjacent Amazonian lowlands.

## Data Availability

All data and code used for our analyses is available from the Dryad Digital Repository: https://doi.org/10.5061/dryad.w0vt4b8wd [103]. Additional tables and figures, data sources and an extended abstract in Spanish are provided in the electronic supplementary material [104].
